# Optimism and Pessimism

**Published:** 1917-05-05

**Authors:** 


### OPTIMISM AND PESSIMISM.

The *Journal of Mental Science* for January last has not been issued so very long, but its exceptional interest compensates for its late appearance. It contains a large number of articles, every one of which is above the average in interest, and some are far above the average. The place of honour is allotted, as is only his due, to the veteran Henry Maudsley, at whose feet we middle-aged and elderly men sat in our youth, and who maintains, and even surmounts, in this performance of his old age, the highest level of his rich maturity. In an article expressed with all his old eloquence, but in which a mild and most telling persuasiveness replaces the fire and scorn of his youth, he discusses and contrasts the respective temperaments and outlooks of the optimist and the pessimist. Truly we may say of the veteran author that his hand has not lost its cunning, his eye is not dimmed, nor is his natural force abated. It would be easy to quote, in fact it is difficult to abstain from quoting, and it would be still more difficult to cease from quoting if once we began. The sentences still flow with their old force, still shine with their old polish. The author is one of the very few medical men now living who is a literary man as well as a medical man, and whose literary style will not suffer by comparison with the best productions of the purely literary artist.

The thesis that Maudsley elaborates is that the optimist pursues and appreciates Joy; the pessimist pursues and appreciates Truth. That exultant optimism springs up afresh in the human breast after every disaster is evidence of an alert and active vitality in a people as well as in a single person. In both it is the effect of life instant and insistent to assert and increase itself; constantly expressed in hope, which, though it springs eternal in the human breast and is never satisfied, being unlimited, serves, at least, to lead by a pleasant path to the end of life; many times, too, persists in the last stage of actual dying. Pessimism gains a deeper insight into and a truer hold of the substance beneath the superficial show. It feels and thinks less vividly, but in the end more deeply, less superficially but more solidly. The pessimist observes sincerely, thinks fully, and feels deeply, unlike in that respect the optimist, who, exultant in the immediate joy of living, cares not to learn or think on the dismal history of human life through the ages. That the sum of happiness and the sum of misery are pretty evenly balanced is proved by the continuance of the dispute as to which is the greater, and that mankind have continued to go on living may be accepted as evidence that they have felt life to be worth living.

The *Journal of Mental Science* is to be congratulated on having persuaded Dr. Maudsley to emerge for a moment from his retirement, and to show that English medicine can still produce a great literary artist as well as a profound philosopher.

